# Integrating Whey Processing: Ultrafiltration, Nanofiltration, and Water Reuse from Diafiltration

**DOI:** 10.3390/membranes14090191

**Published:** 2024-09-06

**Authors:** Vandré Barbosa Brião, Juliane Mossmann, Bruna Seguenka, Samarah Graciola, Jeferson Steffanello Piccin

**Affiliations:** 1 Post-Graduate Program in Food Science and Technology, University of Passo Fundo, Campus I, L1 Building, BR 285, District São José, 611, Passo Fundo 99052-900, Brazil; 83567@upf.br (J.M.); jefersonpiccin@upf.br (J.S.P.); 2 Post-Graduate Program in Food Engineering, Integrated Regional University of Alto Uruguay and Missions, Avenue Sete de Setembro, 1621, PO Box 743, Erechim 99709-910, Brazil; 100432@upf.br; 3 Post-Graduate Program in Civil and Environmental Engineering, University of Passo Fundo, Campus I, L1 Building, BR 285, District São José, 611, Passo Fundo 99052-900, Brazil; 178902@upf.br

**Keywords:** whey protein, lactose, reverse osmosis, activated carbon, wastewater

## Abstract

This work proposes an integrated production of whey protein concentrate (WPC) and lactose and the recovery of water from diafiltration (DF) steps. Whey protein and lactose can be concentrated using ultrafiltration and nanofiltration, respectively, and both can be purified using DF. However, DF uses three-fold the initial volume of whey. We propose a method to reclaim this water using reverse osmosis and adsorption by activated carbon. We produced WPC with 88% protein and purified lactose (90%), and 66% of the water can be reclaimed as drinking water. Additionally, the reclaimed water was used to produce another batch of WPC, with no decrease in product quality. Water recovery from the whey process is necessary to meet the needs of a dairy refinery.

## 1. Introduction

There have been considerable efforts to develop biorefineries globally. These efforts involve the use of different types of biomasses to produce bioproducts and energy. In the operation of a refinery, a single raw material is used to generate several products. One particularly valuable raw material is milk, which is used to produce dozens of products in multipurpose factories. Each product generates secondary streams of residues, which can be valuable in generating other products. 

Each step of manufacturing must be considered to develop a competitive milk refinery. Cheese production provides a good example of valorization [1]. The mass balance in cheese production reveals that each ton of cheese can generate nine tons of whey [2]. Approximately 52% of the total solids, 94% lactose, 96% soluble proteins, and 38% minerals from the milk remain in whey [3]. Only 40–50% of the total whey is recovered as a valuable product [4].

Ultrafiltration (UF) has been used to recover proteins from whey [5,6,7,8,9]. Whey proteins retained by the UF membrane are valuable as high-value food additives [10], nutraceuticals [11], and therapeutics [12]. In contrast, lactose, salts, and the residues of proteins pass through the membrane into the permeate [4,13]. Thus, the recovery of a byproduct (whey) generates another byproduct, the UF permeate, a secondary stream that mainly comprises whey solids, as lactose represents approximately 70% of the dry matter of whey [2]. The permeate, characterized as a saline waste, is a critical component, since the direct discharge of this salty effluent to the environment disrupts the ecological balance [14]. However, the permeate has a potential value, as the lactose can be used in the food or pharmaceutical industries [13,15]. Lactose is an important nutrient for fermentation media that allows for the manufacture of high-value biological products, and its content in oligosaccharides has potential applications in human nutrition [16]. Thus, an intensive and rational process for the subsequent use of whey is required to recover both lactose and proteins. Castro-Muñoz et al. [17] reported the valorization of agro-wastes and membrane-based separation from this perspective, and whey is a potential waste valorization resource.

Several alternatives have been proposed for the use of permeates. Cuartas-Uribe et al. [18] studied the use of nanofiltration (NF) for the recovery of lactose from the permeate and modeled the passage of ions using the Kedem–Spiegler model. De Souza et al. [19] studied several combinations of microfiltration (MF), UF, ion exchange, and reverse osmosis (RO) to separate and purify lactose from whey to obtain 99.8% pure lactose. Baldasso et al. [20] used electrodialysis for a desalting rate of 86% of the permeate, producing 96% pure lactose. Chandrapala et al. [21] used NF to remove lactic acid from whey and retained 95% of the lactose. Colombo et al. [22] studied the production of biohydrogen using the permeate of whey UF with 1.37–1.93 mol of H_2_ per mol of sugar. Djouab and Aïder [23] produced prebiotic lactulose via isomerization by electroactivation using the permeate of whey UF. All these processes require investment for the installations and can generate side streams. For this reason, we propose the addition of diafiltration (DF) steps after the UF of whey and the NF of the permeate.

DF water is usually added to the retained material in later stages of UF plants with many stages (or loops, as commonly termed in the dairy industry) [24]. Furthermore, a multistage process in the DF mode with water could reduce water consumption and increase the lactose concentration in the product [25]. The addition of DF water can increase the permeation of non-protein species across the membrane, thereby increasing the protein concentration.

Nanodiafiltration can partition low-molecular-weight whey components under various processing conditions, depending on the feed composition [21]. An economic advantage of using DF in UF and NF is the use of the same equipment. On the other hand, several stages of DF increase pure water consumption in dairy plants [26]. These volumes can be as high as 1.5 L of water for every L of whey. De Souza et al. [19] tested one volume of DF water for each volume of whey for the purification of proteins in whey UF. Barba, Beolchini and Veglió [27] identified excessive water consumption in whey DF and proposed a multistage countercurrent DF to reduce water consumption. Cabral et al. [13] used two-fold volumes of water for the DF of proteins. Mossmann, Viganó and Brião [6] used DF for whey with the same volume of water for each volume of whey and generated 85% pure protein. Chandrapala et al. [21] used a combination of NF and DF to separate lactic acid from lactose. A common problem in previous studies has been that the DF mode of UF/NF generates a huge volume of effluent. For this reason, we approached the recovery of this effluent from an integrated point of view for water use.

RO has potential value for water recovery in the food industry [28], as RO produces better water quality than NF [29]. RO has been tested for the polishing of treated dairy wastewater [30], for the recovery of condensates from the evaporation of whey and milk [31], and for the treatment of raw wastewater combined with MF [32]. Additionally, Brião et al. [33] reported the recovery of milk solids from rinse water to produce concentrated milk solids and water for cooling purposes, with a payback of the investments in less than one year. However, in these studies, residual organic matter was present in the permeate, and an additional step was required to remove residual organic matter. Despite the added step, the approach could allow for the broader use of reclaimed water, as the water quality was reportedly similar to that of potable water. For this purpose, adsorption by activated carbon is a good alternative, as it has been tested for the treatment of dairy wastewater with a removal rate of approximately 60% [34]. Suárez et al. [35] recovered water from ultrahigh temperature (UHT) milk processing using activated carbon in batch mode following RO. However, maybe the industrial application of activated carbon requires a continuum process. Thus, the combination of RO and activated carbon in a fixed-bed column can be a suitable alternative for the recovery of DF water.

This study aimed to evaluate the coupled production of protein and lactose from whey using a sustainable approach for water reuse by the recovery of DF water.

## 2. Materials and Methods

### 2.1. Whey

**Figure 1:**
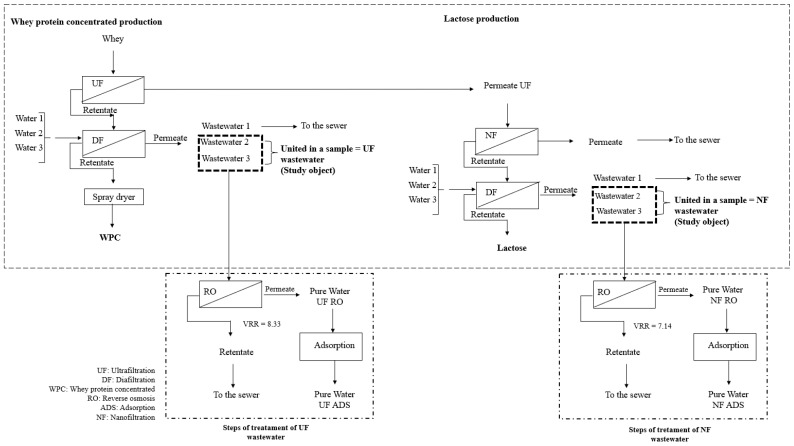
The general schematic of the process of manufacturing WPC by UF + DF, lactose by NF + DF, and the recovery of water from the DF steps by RO + adsorption.

Pasteurized skimmed whey obtained from a mozzarella cheese manufacturing process was kindly donated by Mandaká Alimentos (Boa Vista do Sul, Brazil). We sampled five samples of whey, processed as shown in the flowchart in Figure 1. After separation from the curd, 100 L of pasteurized whey was collected in plastic vessels and immediately transported to the laboratory for processing. We homogenized the samples and they were used for the experiments of UF and NF and kept at 5 °C. The excess was frozen (at −18 °C) to perform the later experiments of water recovery.

### 2.2. Research Strategy

This work followed the schematic shown in Figure 1. The procedure consisted of three steps: (a) the separation of protein by UF + DF and lactose by NF + DF; (b) the treatment of the wastewater from DF by RO using two transmembrane pressures; (c) the post-treatment of the RO by AC; and (d) the production of another batch of whey protein concentrate (WPC) using the reclaimed water from the previous step. Our hypothesis is that it is possible to recover water from the diafiltration steps to produce drinking water.

### 2.3. WPC and Lactose Production

**Figure 2:**
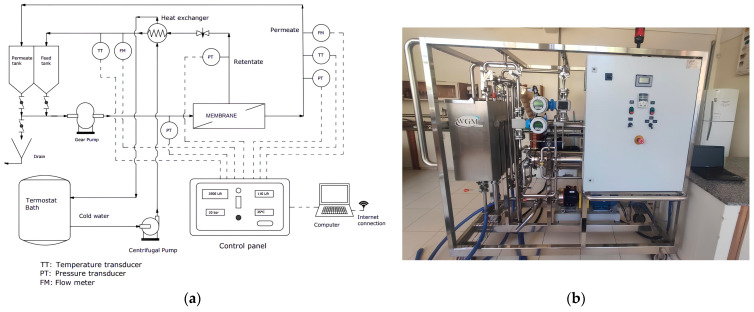
(**a**) Schematic of the pilot rig membrane filtration equipment. (**b**) Picture of the equipment used for the separation and purification of proteins and lactose from whey.

The experiments were conducted in a pilot module provided by WGM Systems (São Paulo, Brazil). Figure 2a shows a schematic of the pilot rig equipment. Figure 2b is a picture of the equipment. The facility consisted of two stainless steel tanks, each with a 150 L capacity. The whey was fed to the tank and driven by a gear pump through the membrane, separating the permeate from the retentate, each of which was driven to one of the two tanks. The retentate passed through a shell heat exchanger on its way to the tank. Cold water (0 °C to 4 °C) was recirculated with the aid of a thermostat bath (100 L capacity, Multi-Pão) to minimize the heat generated by the retentate due to recirculation during the batch operation to maintain the temperature in the range of 5 ± 2 °C. The equipment has transducers for temperature, pressure, and flow meters, which were all connected to a control panel, and a computer to transfer the data via an internet connection.

Whey proteins were separated using UF according to the methods proposed by Baldasso, Barros and Tessaro [36] and adapted by Mossmann, Viganó and Brião [6]. The UF membrane was 2538 KMS-131 (Koch Membrane Systems, Wilmington, MA, USA). The membrane featured a 1.8-inch spiral-wound configuration with an effective filtration area of 1.8 m^2^ and a semipermeable polyethersulfone (PES) layer on a polyester backing material. The molecular weight cutoff (MWCO) of the membrane was 10 kDa. The feed flow rate of whey was adjusted to 1700 L h^−1^ at a pressure of 200 kPa (2 bar) based upon the work of Mossmann, Viganó and Brião [6]. The initial 100 L of whey was reduced to 12 L, representing a volume reduction rate of 8.33.

The UF permeate was subjected to NF to the concentration of the lactose. The same equipment shown in Figure 2 was used, with the replacement of the UF membrane with a NF membrane (2538 SR3, Koch Membrane Systems). This polyamide membrane had a spiral shape and 1.8 m^2^ of filtration area. The hydraulic permeability of the membrane was 6.98 L h^−1^ m^−2^ bar^−1^. The MWCO was 200 Da. UF permeate (88 L) was fed to the equipment. The feed flow rate was adjusted to 1700 L h^−1^ and the pressure was 2000 kPa (20 bar). The permeate was separated until the remaining volume in the feed tank was 12 L (volume reduction rate of 7.33).

In both the UF and NF processes, after the concentration of proteins on UF and lactose on NF, three DF steps were performed to purify the products using the same volume of the initial feed (for each step). Thus, we used 300 L of water for the DF of the proteins and 264 L for the DF of lactose. The water from the second and third DF stages of the protein was combined as “UF Wastewater”. Likewise, the water samples of the second and third lactose DF stages were combined as “NF Wastewater” (Figure 1). The first DF water was purged because of its high chemical oxygen demand (COD). Permeate from a RO membrane was used as DF water because it had an electrical conductivity <10 μS cm^−1^.

Dry WPC was obtained using an LM–MSD 1.0 spray dryer (Labmaq, Ribeirão Preto, Brazil). We fed 500 mL h^−1^ of liquid WPC from the UF + DF step, installed a 2 mm diameter spray nozzle and adjusted the air inlet temperature to 190 °C.

### 2.4. Treatment of the Wastewater from DF by Adsorption Following RO

#### 2.4.1. RO

The equipment shown in Figure 2 was used for the treatment of the DF wastewater by replacing the UF membrane with a model 2538-HRX RO membrane (Koch Membrane Systems). The polyamide membrane had a spiral configuration, 99.3% NaCl rejection, and a filtration area of 1.8 m^2^. We recirculated the permeate and retentate by 1 h and then we concentrated the wastewater until the volume reduction rate was 3.8. Tests were performed at room temperature (23 ± 2 °C). The responses of the experiments were permeate flux and water quality.

In the first step, we tested two transmembrane pressures (1 MPa and 2 MPa, or 10 bar and 20 bar) and evaluated the best condition to proceed to the next step. The responses were the permeate flux, chemical oxygen demand (COD), turbidity, and electrical conductivity of the permeate.

#### 2.4.2. Adsorption with Activated Carbon (AC)

After the RO treatment, the water was subjected to fixed-bed adsorption with granular AC (Vetec, Rio de Janeiro, Brazil). The AC was subjected to a preliminary acid pre-treatment, as suggested by Chern and Chien [37]. The AC (100 g) was washed with 200 mL of a 1 mol L^−1^ HCl solution at 50 °C for 30 min. The AC was rinsed 10 times with 300 mL of boiling deionized water. After rinsing, the AC was dried in an oven at 105 °C for 12 h and then sieved. The fraction retained in the 16 mesh tammy (1.0 mm) was used.

The adsorption system consisted of an infusion pump that was fed a borosilicate glass column (25 mm internal diameter) using a fixed bed of 15 cm height and fed superficial flow rate of 1 cm min^−1^ (4.9 mL min^−1^), as suggested by Dotto et al. [38]. The treated water was collected from the top of the column in a buffer tank. The samples were collected twice, before the adsorption operation and at the end of 38 h of adsorption, for both UF and NF wastewaters.

### 2.5. Production of Another Batch of WPC Using the Reclaimed Water

As the reclaimed water from DF has sufficient quality, we performed another round of experiments in two replicates to produce WPC, to evaluate the hypothesis that water quality interferes with product quality. For this, we repeated the WPC production, but replaced the DF water with the reclaimed water. Further, we analyzed the physicochemical and microbiological characteristics of dried WPC obtained from this stage.

### 2.6. Analytical Methods

The whey and whey products were characterized following the standard protocols of the Association of Official Analytical Chemists [39]. Water characterization followed the standard methods of the American Public Health Association [40]. Table 1 shows the principle and the reference of the methods. Quantification of *Escherichia coli* and total coliforms was performed using Petrifilm plates (3M, Maplewood, MN, USA).

L-lactate was quantified by liquid chromatography coupled to mass spectrometry in series (LC-MS/MS) using Shimadzu equipment, as adapted from Chuang et al. [41]. The reverse phase of the LC-MS/MS system was used with an XR-ODS III/Shimadzu (150 × 2.0 mm × 2.0 μm) analytical column. Mobile phase A consisted of water with 0.1% trifluoroacetic acid. Mobile phase B consisted of methanol in the isocratic mode. The mobile phase flow rate was 0.3 mL min^−1^, the injection volume was 10 μL, and electrospray ionization was used. The triple quadrupole mass analyzer operated in the MS/MS mode. The chromatography analysis time was 3 min at a column temperature of 40 °C, a capillary voltage of 4.5 kV, a desolvation temperature of 400 °C, a desolvation gas flow (N2) of 600 L h^−1^, a spray flow rate of 80 L h^−1^, a collision gas (argon) flow rate of 0.10 mL min^−1^, and a source temperature of 150 °C. Serial MS was used in SRM scan mode and electron ionization in the positive mode. For these optimizations, an analytical solution with a concentration of 250 μg L^−1^ was used. From these injections, we defined the characteristic ions of each compound studied. They were monitored in the SRM mode in quadrupole (Q1) and the ion product was scanned in quadrupole (Q3).

## 3. Results and Discussion

### 3.1. WPC Production

**Figure 3:**
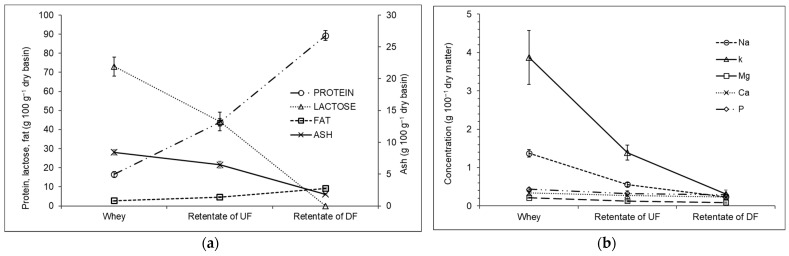
Concentration of protein, lactose, fat, ash (**a**) and salts (**b**) of whey, retentate, and diafiltrated streams of the production of whey protein concentrate by UF +DF.

The main observation from Figure 3a is that as the protein concentration rises, the salts and lactose decrease. In general, both the lactose and ashes are smaller than the pores of UF membranes, and rejections are in a range of 18–33% [5,6]. However, there is protein adsorption on the membrane surface and a cake layer is formed, and ashes and lactose can make up this cake [6]. Thus, the DF step is necessary to wash the cake layer to improve its passage through the membrane. The process heightened the protein concentration by 78.8%, with 37.1% in the UF of whey and 47.7% in the DF of retentate (protein rejection rate 93.9 ± 2.8%). Lactose was reduced to undetectable levels, with an approximately 50% reduction in each step of purification (47.7% for UF and 52.3% for DF). The process also reduced the ash content in the WPC by 70.2%, with DF accounting for most of the reduction (46.8%). The removed salts were also monitored during the UF and DF purification steps. As expected, we observed a high removal of monovalent salts, such as sodium and potassium. Sodium is the second most prevalent mineral in whey. The combination of UF and DF removed 89% of this salt.

The combined use of UF and DF reduced the lactose and salts in whey and increased the protein concentration. Rektor and Vatai [42] studied the combined use of UF and DF to produce protein from mozzarella cheese and concluded that an integrated process is more environmentally friendly and efficient. Baldasso, Barros, and Tessaro [36] studied several strategies involving the addition of water to DF to increase protein purity in the WPC. They concluded that small DF volumes added several times are more effective than the addition of larger volumes of water. A similar procedure was performed by Baldasso et al. [20], where UF was used to concentrate whey protein and four steps of DF were used to purify the proteins. The protein was concentrated 3.8 times (70% on a dry weight basis). In the present study, protein was concentrated 5.1 times (88% on a dry weight basis). The difference in the prior and present results is based on the lactose concentration. We did not detect lactose in the WPC, while Baldasso et al. [20] reported 15% lactose (on a dry weight basis).. De Souza et al. [19] tested several MF and UF protocols for the separation of proteins and purification of lactose from whey. DF was used as a strategy for the purification of lactose, with a ratio of 1 L of water per L of whey. DF is a good strategy to enhance purification. In addition, the same equipment can be used for UF and DF. However, the large volume of water that must be used for purification is problematic, even though DF was responsible for an increase of 47% in the protein in the product. For this reason, we proposed the use of RO and AC to recover water.

Table 2 shows the characteristics of whey and WPC produced. Lactose was undetectable in the WPC. The product had an average of 87% protein and only 2% ash. The reduction in calcium and phosphor was an undesirable characteristic of this process, since these minerals are important for bone health. Another characteristic of this process is the strong reduction in sodium. This could be advantageous in the food industry, where the development of products with low sodium content is a priority.

**Figure 4:**
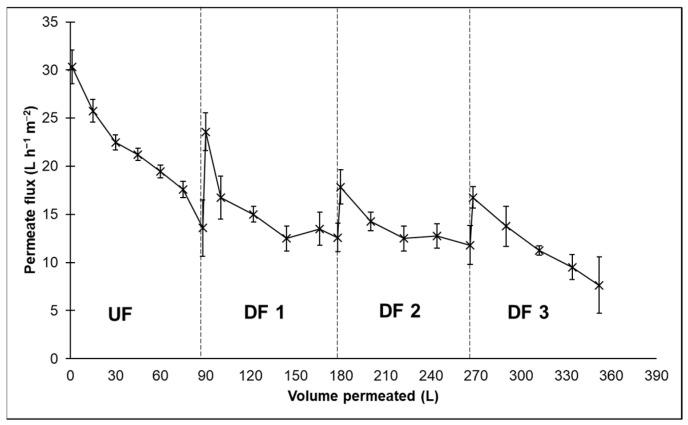
Permeate flux of ultrafiltration (UF) membranes (10 kDa) during the production of whey protein concentrate by UF followed by three diafiltration (DF) steps in pressure of 200 kPa.

UF membrane displayed a strong decay in the permeate flux during the concentration of whey proteins (Figure 4). Compared to the initial volume, the whey was 8.33-fold more concentrated after UF. After this step, the flux was only 45% of the initial value. Further dilution with DF water promoted an increase in flux. At the end of DF, the permeate flux was 26% of the initial value. The permeate flux is reportedly low in whey UF, ranging from 8 to 21 L h^−1^ m^−2^, as shown in previous studies [5,6,20].

Figure 4 shows that at the beginning of the steps of DF, permeate flux increased due to the dilution of the concentrate. However, after the second and third steps of DF, the complete recovery of the initial flux was not observed. This indicated the contribution of reversible and irreversible fouling. Brião et al. [5] showed that irreversible fouling occurs in only 10 s and, in sequence, the formation of the cake layer begins. However, the cake layer is responsible for the majority of the filtration resistance. Cake formation in whey UF has been attributed mainly to proteins and the precipitation of calcium phosphate on the gel layer on the membrane surface [43]. Thus, calcium and phosphorus are rejected in the whey UF and contribute to the declined flux. Additionally, proteins form aggregates by the linkage of free sulfhydryl groups [44], contributing to the growth of the cake. The UF membrane is not a perfect barrier for protein rejection. Small peptides and native proteins can pass through the membrane into the permeate during UF and DF. Consequently, some loss of protein occurs during the process [45].

### 3.2. Lactose Production

**Figure 5:**
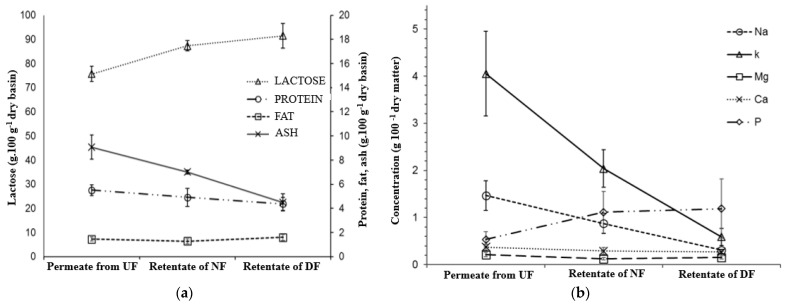
Concentration of lactose, protein, fat, ash (**a**) and salts (**b**) of the permeate from ultrafiltration (UF), retentate and diafiltrate (DF) streams of the production of lactose by nanofiltration (NF) + DF.

The permeate from the UF + DF procedure was fed to the NF membrane. The production of lactose by DF following NF (Figure 5a) led to increased lactose concentration from 75% to 90% (on a dry weight basis). Lactose rejection by the NF membrane was 98.85 ± 2.43% and the salt rejection was only 17.44 ± 1.81%. Cuartas-Uribe et al. [18] tested NF in a continuous DF mode to evaluate lactose separation from whey UF permeate. They reported that the lactose rejection rate ranged between 92% and 96%. For non-charged solutes, such as lactose, sieving by size is the main mechanism of separation of whey constituents by NF membranes, and values can vary between 92% and 98% for different membranes [21]. We did not detect the concentration of protein during the combined use of NF and DF, because approximately 98% of the nitrogen transmitted through the membrane is a non-protein nitrogen species [21].

Monovalent salts (potassium and sodium) were decreased, while bivalent salts (magnesium and calcium) were maintained at a constant value (Figure 5b). At the same time, the concentration of phosphorus increased from 0.54% to 1.12%. The decrease in salts in NF was 22% prior to DF and was 50% after three steps of DF. The desalination of whey by NF is limited (38%), but it can reach 45% after one step of DF [45]. As expected, the concentration of monovalent salts decreased by approximately 80%, while the concentrations of calcium and magnesium remained the same throughout the process. At neutral pH, the precipitation of calcium phosphate was detected upon the NF of the permeate from whey ultrafiltration. A previous study reported that both salts are retained on the membrane surface and contribute to membrane fouling and a decrease in permeate flux [46], and, thus, both calcium and phosphorus do not permeate easily through the membrane.

The characterization of the permeate from UF and purified lactose are presented in Table 3. The concentration increased to 12% lactose as the salts permeated through the NF membrane. The purity of the product exceeded 90%, but proteins and fats were still present. Despite protein being a valuable constituent in whey, protein represented only 15% of the total solids of whey (Table 2). Thus, the permeate from UF must be reclaimed. NF + DF appears to be a rational strategy, as it can concentrate lactose and reduce salts from this stream. Some companies just dry the permeate in a spray dryer, but the product contains 10% salts (Table 3) and has low commercial value. De Souza et al. [19] suggested desalinating the permeate obtained from UF by ion exchange (IE) to increase the lactose purity, and the process of UF + DF + IE achieved 0.5% ash and 98% lactose. The use of NF + DF before IE could extend the cycle of the production/regeneration of resins. Other membrane processes were tested to determine the concentration of the whey UF. Membrane distillation was tested for the concentration of the permeate from the UF of whey by Kezia et al. [14]. The authors reported a final concentration of 30% (*w*/*w*) of total solids. The water recovery ranged from 37% to 83%. However, both salts and lactose were concentrated, and the salinity of the final product was high. Thus, it had low commercial value. For a viable process, we believe that the concentration of lactose is necessary, with the simultaneous desalination of lactose by NF + DF.

**Figure 6:**
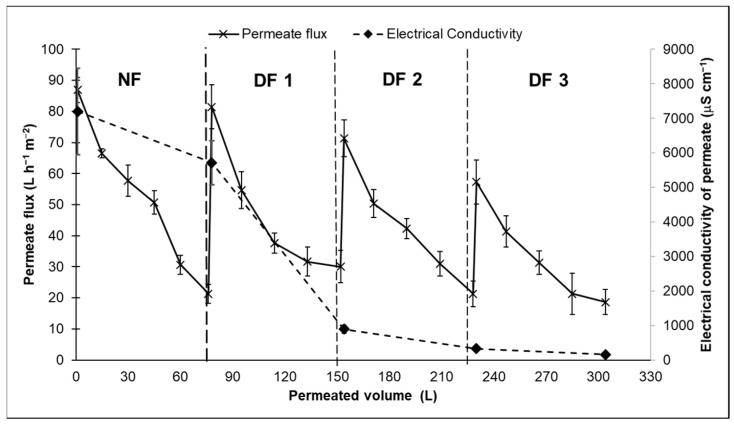
Permeate flux of nanofiltration (NF) membrane (200 Da) and electrical conductivity of the permeate during the production of lactose by NF followed by three steps of diafiltration (DF) at 20 bar and volume reduction rate of 7.3.

The permeate flux of the NF membrane exhibited a strong decay during the concentration and purification of lactose (Figure 6). Similar behavior was previously described using the NF HL membrane manufactured by Osmonics [21]. The authors concentrated the initial volume from 85 L to 15 L, representing a reduction rate of 5.6. At the beginning of NF, the flux was 89 L h^−1^ m^−2^. The permeate flux was approximately 20 L h^−1^ m^−2^ at the end of NF and the three steps of DF. Another interesting observation concerned the initial flux during DF. After dilution with water for DF, the permeate flux did not completely recover. This indicates the presence of irreversible fouling on the membrane caused by residues of protein and by the precipitation of calcium phosphate. The deposit is easily removed by acid cleaning [47]. We also monitored the electrical conductivity (EC) of the permeate during the process to determine whether DF could be completed. The first DF step reduced the EC by 84%. After the third DF step, the EC of the permeate was only 158 μs cm^−1^. At this time, the EC of the retentate was 2814 μs cm^−1^ (Table 4). Thus, the transmission of salts through the membrane was only 5.6%. We decided to finish the lactose purification. NF + DF reduced sodium and potassium. However, as discussed earlier, magnesium, calcium, and phosphorus were retained by the membrane, and there was no further reduction in EC.

**Figure 7:**
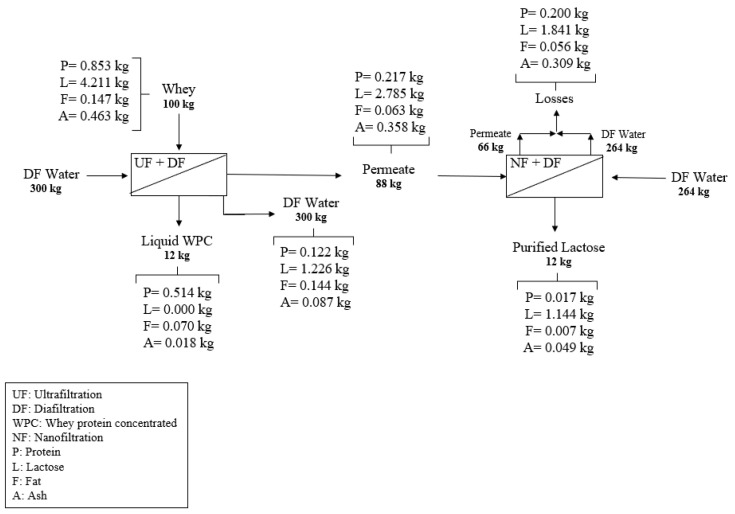
Mass balance of the integrated whey processing.

Figure 7 shows the mass balance of the integrated process. We recovered 60% of protein from whey in the WPC stream. Protein is a valuable product in the integrated process, but there are losses in the purification by DF and through the permeate of ultrafiltration. On the other hand, the process recovered only 1.144 kg of lactose from an initial mass of 4.211 kg (27%). We observed 29% (1.226 kg) of lactose losses only in the DF water in the UF + DF. Future studies can include the separation of both protein and lactose in this water, aiming for the best efficiency of the process.

### 3.3. Water Recovery from DF

**Figure 8:**
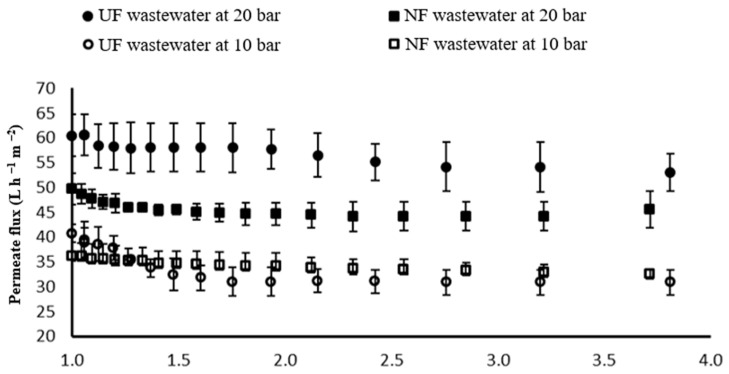
Permeate flux of reverse osmosis treating the wastewater from the diafiltration of whey protein concentrate (WPC) and lactose along the volume reduction rate.

For integrated whey processing, we proposed the recovery of wastewater from the DF steps by RO and adsorption by AC. Figure 8 shows the permeate flux of the RO membrane of wastewater from the DF of WPC and lactose production. Both curves displayed a slight decline in the permeate flux (9%) after 13 min of permeation. However, the wastewater from the DF of lactose was more contaminated with higher COD (Table 4 and Table 5). Thus, it had a lower permeate flux.

The data of the characterization of wastewater from the DF of protein at the UF membrane and the water treated by RO and RO + AC are presented in Table 4. RO removed 99.0% of the COD. After the adsorption of AC, the COD removal was 99.5%. A similar result was observed in the characterization of wastewater from the DF of lactose and the water treated by RO and RO + AC (Table 5). RO removed 99.7% of the COD. After the AC step, a removal of 99.9% was obtained. The data of Table 4 and Table 5 demonstrate the essential role of RO in this process. RO membranes also displayed a high rejection rate of COD in previous studies of the RO treatment of dairy wastewater [33,48]. However, these studies also observed traces of organic matter in the permeate, and so a low COD concentration remained in the permeate. This prevents the use of this water inside the factory because the food industry requires potable quality for cleaning procedures.

In addition, the RO permeate still smells like “cooked milk”. Suárez et al. [35] used RO + AC to purify water from the UHT milk process. The AC was able to polish the effluent and remove 100% of the COD. However, the water still had this odor. Traces of volatile organic compounds come from milk fat and some protein degradation, and their presence in milk is induced by heat treatments like evaporation. Vourch et al. [49] reported that organic molecules involved in the COD of wastewater from evaporated milk also include small molecules, such as lactate, ethanol, acetone, acetoin, diacetyl, and dimethyl sulfide. All these small volatile molecules can contribute to the COD. Their volatile character contributes to the “cooked milk” odor. We did not detect this odor in our experiments because the DF wastewater was not subjected to intense heat treatment. Thus, AC polishes water to remove organic residues. We chose to evaluate L-lactate as a representative of these small constituents, since whey was not subjected to evaporation and L- lactate is a form of lactic acid. Even after RO, residual lactate was still present in the water (removal of 96.5–97.5%, Table 4 and Table 5). However, after the AC step, 99.8% of the lactate was removed.

**Figure 9:**
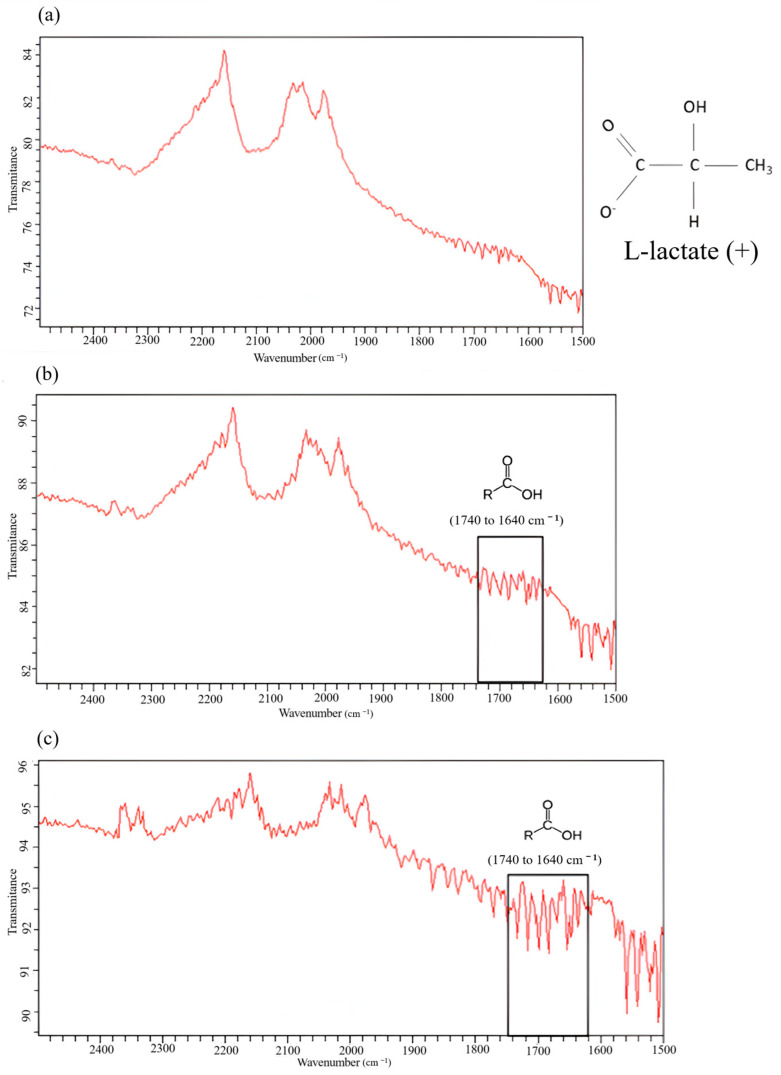
Fourier transform–infrared spectra of (**a**) virgin activated carbon and (**b**) activated carbon (AC) after the passage of UF wastewater treated by reverse osmosis (RO), and (**c**) AC after the passage of NF wastewater treated by RO.

We performed Fourier transform–infrared analysis of the AC before and after the treatment of the wastewater (Figure 9). Prominent peaks (in red line) of the band between 1640 cm^−1^ and 1640 cm^−1^ were evident (Figure 9b,c). Skoog et al. [50] attributed this range to the stretching of C=O bonds (1690 cm^−1^ and 1760 cm^−1^) of lactic acid. Thus, after the water treatment, the AC step resulted in prominent peaks of C=O bonds, likely due to lactate adsorption onto the superficial matrix of AC.

The pH of the water was reduced after passage through the AC (Table 4 and Table 5). We performed an acid treatment in the AC before use. The pH of the water treated by AC following RO was lower than the initial value. Additionally, AC heightened the EC of the water. However, this is not a negative finding, as the correction of pH is easily handled by adding alkali agents, such as NaOH or Na_2_CO_3_.

**Figure 10:**
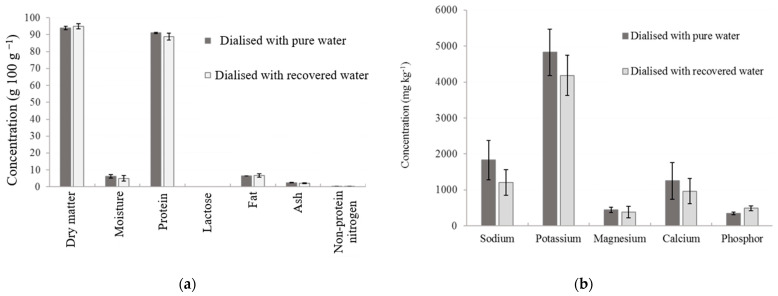
Comparison between whey protein concentrate prepared by dialysis with pure water and reclaimed water by reverse osmosis (RO) + activated carbon (AC) concerning (**a**) macroconstituents and (**b**) salts.

We compared the water quality treated by RO + AC, with some possible uses in the whey factory (Table 6). The data concerning the low organic matter (total organic carbon and biological oxygen demand) and turbidity, the absence of color and *E. coli*, hardness, and EC showed that the water was usable for the three destinations of drinking water, cooling towers and boilers. The correction of pH must be carried out to adjust to a neutral range for the three destinations. On the one hand, the use of reclaimed water in cooling towers and boilers can be easily accepted by managers and regulated. On the other hand, the acceptance of the use of this reclaimed water for drinking purposes can be a barrier, because we have to be sure of the water quality and possible side effects on food safety. We believe that the latter use is possible. To this end, we produced WPC powder by replacing the water of DF with the reclaimed water by RO + AC and evaluated the quality of the powder (Figure 10). A statistical analysis revealed no difference between the products, even though the reclaimed water contained dissolved salts as evidenced by the EC of 43–60 µS cm^−1^ (Table 6). A microbiological analysis detected <100 colony-forming units (CFU) g^−1^ for both products (the acceptable limit is a maximum of 30,000 CFUs g^−1^ in Brazilian regulations). Thus, both physicochemical and microbiological qualities have been assured for the use of reclaimed water for the use of DF for WPC production. It is possible to use reclaimed water treated using RO + AC from DF as safe water for whey processing. This will require strict control of both RO and AC processes, as well as an effective and efficient water quality monitoring program. This could overcome what has been the main barrier in the paradigm for the use of water in the dairy industry. Strict oversight will be needed to guarantee the food safety of the products.

Leverenz, Tchobanoglous, and Asano [51] reported the need for the direct reuse of water, as this water quality is safe. We agree with the authors, but the water safety must be ensured. The water balance of our process (Figure 7) revealed that for 100 L of whey fed to the integrated production of protein and lactose, a total volume of 564 L of water is required to perform the three steps of DF of protein and lactose. The predicted recovery of water is 376 L, representing 66% of the water used for DF. For example, one dairy industry produces 10 tons d^−1^ of cheese and generates approximately 90 tons d^−1^ of whey. Thus, using Figure 7 as a reference, this industry can produce 462 kg of WPC and 1002 kg of lactose. However, this company will use 507 m³ of water for diafiltration. This huge volume of water can prevent the operation of the industry. However, our process can reuse 66% of this volume (335 m^3^) and make the company more sustainable. However, the company must ensure the quality of the reclaimed water as drinking water, and, thus, this is a good strategy for whey processing.

**Table 6:** Physical chemical and microbiological characteristics of recovered water compared to current legislation.

Parameters	Wastewater from DF of Protein Treated by RO + AC	Wastewater from DF of Lactose Treated by RO + AC	Drinking Water ^1^	Make Up of Cooling Towers ^2^	Feeding Boilers ^3^
COD (mg L^−1^)	2.5	1.4	NS	NS	NS
TOC (mg L^−1^)	<1.0	<1.0	<5	NS	<1
Color (Hz)	0	0	Acceptable to consumers	NS	NS
Turbidity (NTU)	0.10	0.12	1	NS	NS
pH	4.00	3.90	6.5–9.5	6.0–9.0	8.3 < pH < 10
Electrical conductivity (µS cm^−1^)	60.7	43.0	2500	NS	10
Hardness (mg L^−1^)	<1	<1	NS	NS	<1
BOD5	3	3	NS	<30	NS
*Escherichia coli*	Absence	Absence	Absence	NS	NS
Total coliforms (CFU/100 mL)	Absence	Absence	Only for monitoring	≤200	NS

^1^ The European Parliament and the Council of the European Union [52]. ^2^ Adapted from the United States Environmental Protection Agency [53]; ^3^ American Boiler Manufacturers Association [40], with a pressure < 450 psig.

## 4. Conclusions

This work focused on the integrated production of protein and lactose in whey by UF and NF, respectively, followed by three steps of DF. Coupled production is a good example of processing that matches the concept of a dairy refinery. The WPC had a protein concentration of 87% in the dry product, and the lactose produced showed a concentration of 91% in the dry matter. 

The proposed strategy to recover DF water by adsorption following RO is necessary for the sustainable production of both products. The reclaimed water has potable quality, as RO can remove most of the organic matter. Adsorption by AC polishes water with lower organic material, such as lactate and odor. We used 300 L of DF water for 100 L of initial whey and 264 L of DF water for 88 L of permeate from UF. The method recovered 376 L (66%) of the DF water.

The DF of whey protein with this water produced a product with the same physicochemical and microbiological characteristics as the traditional protein produced with pure water for the DF steps. However, the use of this water as drinking water depends on several factors, such as regulation and strict control to guarantee the food safety of the products.

## Figures and Tables

**Table 1:** Analytical methods for characterization of whey products and water from diafiltration.

Parameter	Method	Reference Number
pH	Bench pH meter	981.12 [39]
Electrical conductivity	Bench conductivimeter	2510B [40]
Dissolved solids (° Brix)	Pocket meter of dissolved solids	932.12 [39]
Dry matter	Drying in oven at 105 °C	925.09 [39]
Moisture	Drying in oven at 105 °C	925.09 [39]
Protein	Kjeldahl nitrogen	988.05 [39]
Lactose	Reducing sugars by Lane–Eynon method	980.13 [39]
Fat	Gerber or extraction by Soxhlet method	989.05 or 920.39 [39]
Ash	Burning in oven at 550 °C	942.05 [39]
Non-protein nitrogen	Previous precipitation of proteins with trichloroacetic acid (TCA) and later nitrogen analysis by Kjeldahl method	976.13 [39]
Sodium	Flame emission spectroscopy	975.03 [39]
Potassium	Flame emission spectroscopy	975.03 [39]
Magnesium	Atomic Absorption Spectroscopy	985.01 [39]
Calcium	Atomic Absorption Spectroscopy	970.08 [39]
Phosphor	Visible Spectroscopy	965.17 [39]
Chemical Oxigen Demand	Titration method	5220 D [40]
Turbidity	Visible Spectroscopy	2130 B [40]
Color	Visible Spectroscopy	2120 B [40]

**Table 2:** Characterization of whey and diafiltered whey protein concentrate (WPC).

Parameter	Liquid Whey	Dry WPC
pH	6.29 ± 0.17	NA
Electrical conductivity (mS cm^−1^)	5.55 ± 1.11	NA
Dissolved solids (° Brix)	6.00 ± 0.20	NA
Dry matter (g 100 g^−1^)	5.49 ± 0.98 ^a^	98.16 ± 1.09 ^b^
Moisture (g 100 g^−1^)	94.51 ± 0.88 ^a^	1.84 ± 0.89 ^b^
Protein * (g 100 g^−1^)	15.53 ± 2.08 ^a^	88.82 ± 7.38 ^b^
Lactose * (g 100 g^−1^)	73.01 ± 4.53	ND
Fat * (g 100 g^−1^)	2.68 ± 0.16 ^a^	9.11 ± 0.73 ^b^
Ash * (g 100 g^−1^)	8.43 ± 2.00 ^a^	2.02 ± 0.35 ^b^
Non-protein nitrogen * (g 100 g^−1^)	0.06 ± 0.02 ^a^	0.05 ± 0.02 ^b^
Sodium * (g 100 g^−1^)	1.34 ± 0.38 ^a^	0.23 ± 0.02 ^b^
Potassium * (g kg^−1)^	3.87 ± 0.39 ^a^	0.31 ± 0.06 ^b^
Magnesium * (g kg^−1^)	0.21 ± 0.04 ^a^	0.09 ± 0.04 ^b^
Calcium * (g kg^−1^)	0.34 ± 0.10 ^a^	0.23 ± 0.01 ^b^
Phosphor * (g kg^−1^)	0.44 ± 0.80 ^a^	0.29 ± 0.03 ^b^

^a,b^ Results of four replicates ± standard deviation in the same row. Identical indices represent equal values in the statistical comparison of mean values when *p* < 0.05. ND: not detected by the analytical method; NA: not applicable; * dry matter.

**Table 3:** Characterization of the permeate from ultrafiltration (UF) and of the diafiltration (DF) retentate of the nanofiltration (NF-DF).

Parameter	Permeate of UF	Retentate of NF-DF
pH	6.15 ± 0.05 ^a^	6.52 ± 0.20 ^b^
Electrical conductivity (mS cm^−1^)	4.00 ± 0.40 ^a^	2.81 ± 0.56 ^b^
Dissolved solids (° Brix)	5.05 ± 1.44 ^a^	10.98 ± 1.10 ^b^
Dry matter (g 100 g^−1^)	4.98 ± 0.19 ^a^	10.03 ± 1.00 ^b^
Moisture (g 100 g^−1^)	95.52 ± 1.19 ^a^	89.72 ± 1.22 ^b^
Protein * (g 100 g^−1^)	5.90 ± 1.45 ^a^	4.09 ± 0.94 ^a^
Lactose * (g 100 g^−1^)	80.01 ± 3.13 ^a^	90.05 ± 5.20 ^b^
Fat * (g 100 g^−1^)	1.45 ± 0.37 ^a^	1.49 ± 0.39 ^a^
Ash * (g 100 g^−1^)	10.96 ± 1.00 ^a^	4.05 ± 0.23 ^b^
Non-protein nitrogen * (g 100 g^−1^)	0.78 ± 0.33 ^a^	0.32 ± 0.05 ^a^
Sodium * (g 100 g^−1^)	1.47 ± 0.31 ^a^	0.31 ± 0.02 ^b^
Potassium * (g 100 g^−1^)	4.05 ± 0.05 ^a^	0.51 ± 0.08 ^a^
Magnesium * (g 100 g^−1^)	0.21 ± 0.04 ^a^	0.15 ± 0.06 ^a^
Calcium * (g 100 g^−1^)	0.37 ± 0.07 ^a^	0.27 ± 0.04 ^b^
Phosphor * (g 100 g^−1^)	0.54 ± 0.16 ^a^	1.12 ± 0.07 ^a^

^a^ Results of four replicates ± standard deviation in the same row. Identical indices represent equal values in the statistical comparison of mean values when (*p* < 0.05). ^b^ ND: not detected by the analytical method; * dry matter.

**Table 4:** Physical chemical characteristics of the UF wastewater and after the proposed treatment by reverse osmosis (RO) and activated carbon (AC).

Parameters	Wastewater from DF of WPC ^a^	Wastewater from DF of WPC Treated by RO ^b^	Wastewater from DF of WPC Treated by RO + AC ^c^
COD (mg L^−1^)	539.4 ± 111.1	5.3 ± 1.1	2.5 ± 0.2
TOC (mg L^−1^)	366.9 ± 0.3	<1.0	<1.0
Color (Hz)	5.0 ± 1.0	ND	ND
Turbidity (NTU)	3.2 ± 0.1	0.1 ± 0.0	0.1 ± 0.0
pH	5.20 ± 0.07	5.34 ± 0.05	4.00 ± 0.07
Conductivity (µS cm^−1^)	197.8 ± 8.6	23.7 ± 4.1	60.7 ± 0.9
L-Lactate (mg L^−1^)	36.62 ± 5.80	0.90 ± 0.01	0.05 ± 0.01

^a^ Wastewater from the 2nd and 3rd stages of the diafiltration of protein. ^b^ Wastewater from the 2nd and 3rd stages of protein diafiltration after reverse osmosis. ^c^ Wastewater from the 2nd and 3rd stages of protein diafiltration after reverse osmosis and adsorption in activated carbon. ND: Not detected by the analytical method.

**Table 5:** Physical chemical characteristics of the NF wastewater and after the proposed treatment by reverse osmosis (RO) and activated carbon (AC).

	Wastewater Generated by DF of Lactose ^a^	Wastewater from DF of Lactose Treated by RO ^b^	Wastewater from DF of Lactose Treated RO + AC ^c^
COD (mg L^−1^)	1591.4 ± 53.0	3.8 ± 0.5	1.4 ± 0.2
TOC (mg L^−1^)	277.2 ± 0.1	<1.0	<1.0
Color (Hz)	24.0 ± 0.9	ND	ND
Turbidity (NTU)	3.9 ± 0.1	0.1 ± 0.0	0.1 ± 0.0
pH	5.34 ± 0.05	5.60 ± 0.05	3.90 ± 0.05
Conductivity (µS cm^−1^)	302.1 ± 6.6	27.7 ± 1.0	43.0 ± 1.1
L-Lactate (mg L^−1^)	24.79 ± 1.27	0.9 ± 0.01	0.04 ± 0.01

^a^ Wastewater from the 2nd and 3rd stages of diafiltration of protein. ^b^ Wastewater from the 2nd and 3rd stages of protein diafiltration after reverse osmosis. ^c^ Wastewater from the 2nd and 3rd stages of protein diafiltration after reverse osmosis and adsorption in activated carbon. ND: Not detected by the analytical method.

## Data Availability

The data presented in this study are available in the patent regarding the production of protein/lactose, patent number BR 102016024530-3. These data were derived from the following resources available in the public repository of the Brazilian thesis: http://tede.upf.br/jspui/handle/tede/1472, and http://tede.upf.br/jspui/handle/tede/1349. Accessed on 4 September 2024.
